# Running-Related Biomechanical Risk Factors for Overuse Injuries in Distance Runners: A Systematic Review Considering Injury Specificity and the Potentials for Future Research

**DOI:** 10.1007/s40279-022-01666-3

**Published:** 2022-03-05

**Authors:** Steffen Willwacher, Markus Kurz, Johanna Robbin, Matthias Thelen, Joseph Hamill, Luke Kelly, Patrick Mai

**Affiliations:** 1 Department for Mechanical and Process Engineering, Offenburg University of Applied Sciences, Offenburg, Germany; 2 Department of Quality Technology & Mechanical Engineering, Mid Sweden University, Östersund, Sweden; 3 Institute for Biomechanics and Orthopaedics, German Sport University Cologne, Cologne, Germany; 4 Biomechanics Laboratory, University of Massachusetts, Amherst, MA USA; 5 School of Human Movement and Nutrition Sciences, University of Queensland, St. Lucia, QLD Australia

## Abstract

**Background:**

Running overuse injuries (ROIs) occur within a complex, partly injury-specific interplay between training loads and extrinsic and intrinsic risk factors. Biomechanical risk factors (BRFs) are related to the individual running style. While BRFs have been reviewed regarding general ROI risk, no systematic review has addressed BRFs for specific ROIs using a standardized methodology.

**Objective:**

To identify and evaluate the evidence for the most relevant BRFs for ROIs determined during running and to suggest future research directions.

**Design:**

Systematic review considering prospective and retrospective studies. (PROSPERO_ID: 236,832).

**Data Sources:**

PubMed. Connected Papers. The search was performed in February 2021.

**Eligibility Criteria:**

English language. Studies on participants whose primary sport is running addressing the risk for the seven most common ROIs and at least one kinematic, kinetic (including pressure measurements), or electromyographic BRF. A BRF needed to be identified in at least one prospective or two independent retrospective studies. BRFs needed to be determined during running.

**Results:**

Sixty-six articles fulfilled our eligibility criteria. Levels of evidence for specific ROIs ranged from conflicting to moderate evidence. Running populations and methods applied varied considerably between studies. While some BRFs appeared for several ROIs, most BRFs were specific for a particular ROI. Most BRFs derived from lower-extremity joint kinematics and kinetics were located in the frontal and transverse planes of motion. Further, plantar pressure, vertical ground reaction force loading rate and free moment-related parameters were identified as kinetic BRFs.

**Conclusion:**

This study offers a comprehensive overview of BRFs for the most common ROIs, which might serve as a starting point to develop ROI-specific risk profiles of individual runners. We identified limited evidence for most ROI-specific risk factors, highlighting the need for performing further high-quality studies in the future. However, consensus on data collection standards (including the quantification of workload and stress tolerance variables and the reporting of injuries) is warranted.

**Supplementary Information:**

The online version contains supplementary material available at 10.1007/s40279-022-01666-3.

## Key Points

**Table:** 

Levels of evidence for overuse injury-specific biomechanical risk factors range from conflicting to moderate evidence.
Findings were derived from studies with primarily moderate to high quality.
Running related biomechanical risk factors are injury specific.
Joint mechanics within the frontal and transverse planes are more often related to running overuse injury risk compared to sagittal plane joint mechanics.

## Introduction

Running overuse injuries (ROIs) are widespread, with a reported overall incidence of 19.4–79.3% [1]. Depending on the type of runner, definitions of injury, and follow-up periods, running-related injury incidence rates range between 2.5 and 33.0 injuries per 1000 h of running [2]. The origins of ROIs are complex [3, 4] but principally result from an accumulation of repetitive stress applied to the body without sufficient rest for tissue remodeling, resulting in tissue degeneration [5]. The stress response is a function of both tissue characteristics (influenced by lifestyle and genetic factors) and stress application characteristics (e.g., amplitude, frequency, duration) [6]. However, the non-invasive determination of these stresses is challenging, as is the measurement of stress accumulation in everyday life and sports [7]. To determine structure-specific stresses, computational models need to integrate precise anatomical information (e.g., biological tissues' properties and geometry) and the potential neuromuscular control strategy that governs force and power production [8].

Therefore, researchers and practitioners often attempt to predict injury risk based on less direct, less computational and information-expensive biomechanical parameters as surrogate variables to link running biomechanics and injury risk. Such running-related biomechanical risk factors (BRFs) include kinematic and kinetic parameters derived from ground reaction force, pressure mapping, electromyographic, and motion capture data. Using BRFs, runners at risk of developing an ROI can be identified. However, to prevent ROIs, further knowledge on cause-effect relationships is needed [9].

Within a framework of injury development [10, 11], the most relevant BRFs could serve as a source for the improvement of technical (e.g., running shoes or foot orthoses/insoles), training, and feedback system interventions (e.g., in gait retraining or through "digital coaches" based on wearable sensor information) by targeting populations at risk. Research on BRFs employs different research designs and populations. The wealth of information is challenging to oversee.

ROIs can affect different types of tissues (e.g., tendon or bone) [12] within different anatomical locations, with the knee being the most frequently injured site [1]. Therefore, it is likely that the mechanical factors increasing the likelihood of sustaining an ROI differ for different types of ROIs. However, previous systematic reviews on the topic have either focused on BRFs for sustaining ROIs as a whole or considered only a single ROI. A systematic review applying the same methodology (e.g., inclusion and exclusion criteria) to studies analyzing BRFs concerning specific ROI subgroups is currently missing in the literature.

Therefore, the aim of this review article is: (1) to identify the most relevant BRFs and evaluate their evidence concerning the most prevalent ROIs; and (2) to suggest future directions of research to improve the understanding of the relationship between running biomechanics and overuse injury development while considering the interplay between BRFs, workload characteristics and individual, structure-specific stress tolerances.

## Methods

### Search Strategy and Risk Factor Extraction

In the context of this review, we considered a variable a BRF if it was identified as being different between injured and uninjured runners with a statistical test.

The systematic review aimed to extract the evidence for BRFs for the ROIs with the highest prevalence and incidence. Therefore, based on the work of Lopes et al. [12], we examined BRFs for the following ROIs: medial tibial stress syndrome (MTSS), Achilles tendinopathy (AT), plantar fasciitis (PF), patellar tendinopathy (PT), iliotibial band syndrome (ITBS), tibial stress fracture (TSF), hamstring tendinopathy (HT), and patello-femoral pain syndrome (PFPS). We followed the Preferred Reporting Items for Systematic Reviews and Meta-analyses (PRISMA) guidelines [13]. Before starting the literature review, we registered this study at PROSPERO (record ID 236,832).

We scanned the PubMed database for articles comparing the running biomechanics of injured and uninjured individuals for the eight most common ROIs. For each ROI, we used an injury-specific search string (for details, please refer to the Supplementary Digital Content (SDC1)). In short, each search string comprised combinations of runn* (i.e., the main activity), the injury location (e.g., tibia*), multiple keywords to characterize injury-specific physical complaints (e.g., risk OR tend* OR pain), and the study design (e.g., prospective OR retrospective). We used an additional combination of keywords to obtain original English articles involving human participants. The initial search for ITBS, MTSS, and HT took place on 4 February 2021. One day after (5 February 2021) the search strings for AT, PT, PFPS, PF, TSF were applied. Search results, including titles and abstracts, were uploaded to the web interface of rayyan.qcri.org [14]. We then screened titles and abstracts of the articles using the following criteria:

Inclusion criteria:

- Studies in the English language
- Prospective or retrospective studies addressing at least one of the ROIs of interest and relating injury risk to at least one discrete BRF
- Studies considering discrete kinematic, kinetic (including pressure measurements), or electromyographic BRFs
- The primary sport of the investigated study sample was running.

Exclusion criteria:

- No BRF analyzed
- Studies that sampled from populations where distance running was not the primary sport
- Studies only addressing biomechanical risk factors during dynamic activities other than running (e.g., walking or stair climbing)
- Studies addressing anthropometric factors (e.g., leg alignment, foot posture index) or strength measurements (e.g., toe strength or hip abduction strength)
- Studies including military or physical education students due to the unknown effects of concurrent training
- Studies (obviously) publishing duplicate results from the same subject sample as in a previous publication obtained from the same group; however, if the subsequent publications addressed novel potential BRFs which were not addressed in the first publication, the novel BRFs from the second study were included. Any BRFs that had been reported in the first study were excluded
- Non-original articles (e.g., reviews or conference articles) or articles not written in English.

When considering retrospective studies, we included studies where runners were still suffering from the injury or where they had already recovered from the injury. However, since we were addressing BRFs during running, the participants were all able to run for the data collections. We further considered retrospective studies when runners had recovered from the injury independent from the timeframe during which the injury had occurred, i.e., for how long the runners had already recovered from the injury. Details on these aspects are reported for each study in SDC3.

After applying the inclusion and exclusion criteria, two review team members independently screened titles and abstracts of studies found through the search strategy for potentially relevant studies. The selection of appropriate studies was discussed between the team members, and in the case of disagreements, these were resolved through consultation with another member of the review team. Subsequently, full texts were screened based on the same exclusion and inclusion criteria.

Additional sources were identified through the reference list of the eligible articles from the initial search and a co-citation method using the bibliographic coupling concept (www.connectedpapers.com).

Data on study characteristics were extracted from all included articles by members of the review team. Discrepancies were identified and resolved through discussion (with another reviewer if necessary). This data extraction included publication details (author and year), general information on injury type, specific running population, sample size, data collection method, running speed and footwear used during testing, and biomechanical outcome variables. Furthermore, we determined whether potential risk factors found in other studies could have been calculated based on the reported data collection methods. We also collected data on participant characteristics (e.g., age, sex, height).

### Relevance Criterion for Considering Running-Related Risk Factors

We considered a BRF relevant if at least one prospective study or two retrospective studies from independent data collections found a significantly different value of a BRF for a specific ROI.

### Quality Rating and Risk of Bias Assessment

We followed the same procedure as in a previously published review [15] using selected components from the 'Quality Index' developed by Downs and Black (D&B) [16]. The modified 'Quality Index' scale consists of 15 items. All points of the modified 'Quality Index' were summed to provide a quality score for each study. Studies scoring 11 or greater were considered to be of high quality, studies with scores of six to ten were considered to be of moderate quality, and studies with scores of five or less were considered to be of low quality [17]. Two members of the review team independently assigned all ratings. Outcomes were discussed in a team meeting, and discrepancies between raters were resolved by consulting a third rater.

Due to poor reliability observed in items addressing external validity in the complete D&B Quality Index [16], we performed a separate risk of bias assessment using a 10-point checklist, previously described in a systematic review of ROIs [12]. Each item was rated with either 1, referring to a low risk of bias, or 0, referring to a high risk of bias. If certain items could not be categorized, we assigned them a value of 0. Overall, we summed up the ten items' scores. When less than half of the maximum possible points (i.e., ≤ 5 of 10 possible points) were reached, we considered the study to have a high risk of bias. Again, the rating was assigned independently by two raters. Inconsistencies in any item were first discussed between the two raters. If no consensus between the two raters was achieved, a third rater resolved the conflicts.

To determine the strength of evidence of a BRF for a specific ROI, we followed the same approach as a previous review focussing on the role of BRFs for running injuries in general [18]. These authors used the following categories described in detail by van Tulder et al. [19]:

- Strong evidence: Consistent findings among three or more studies, including a minimum of two high-quality studies.
- Moderate evidence: Consistent findings among two or more studies, including at least one high-quality study.
- Limited evidence: Findings from at least one high-quality study or two low- or moderate-quality studies.
- Very limited evidence: Findings from one low- or moderate-quality study.
- Inconsistent evidence: Inconsistent findings among multiple studies (e.g., one or multiple studies reported a significant result, while one or multiple studies reported no significant result).
- Conflicting evidence: We defined conflicting as contradictory results between studies (e.g., one or multiple studies reported a significant result in one direction, while one or multiple studies reported a significant result in the other direction).
- No evidence: Results were insignificant and derived from multiple studies regardless of quality.

## Results

**Fig. 1:**
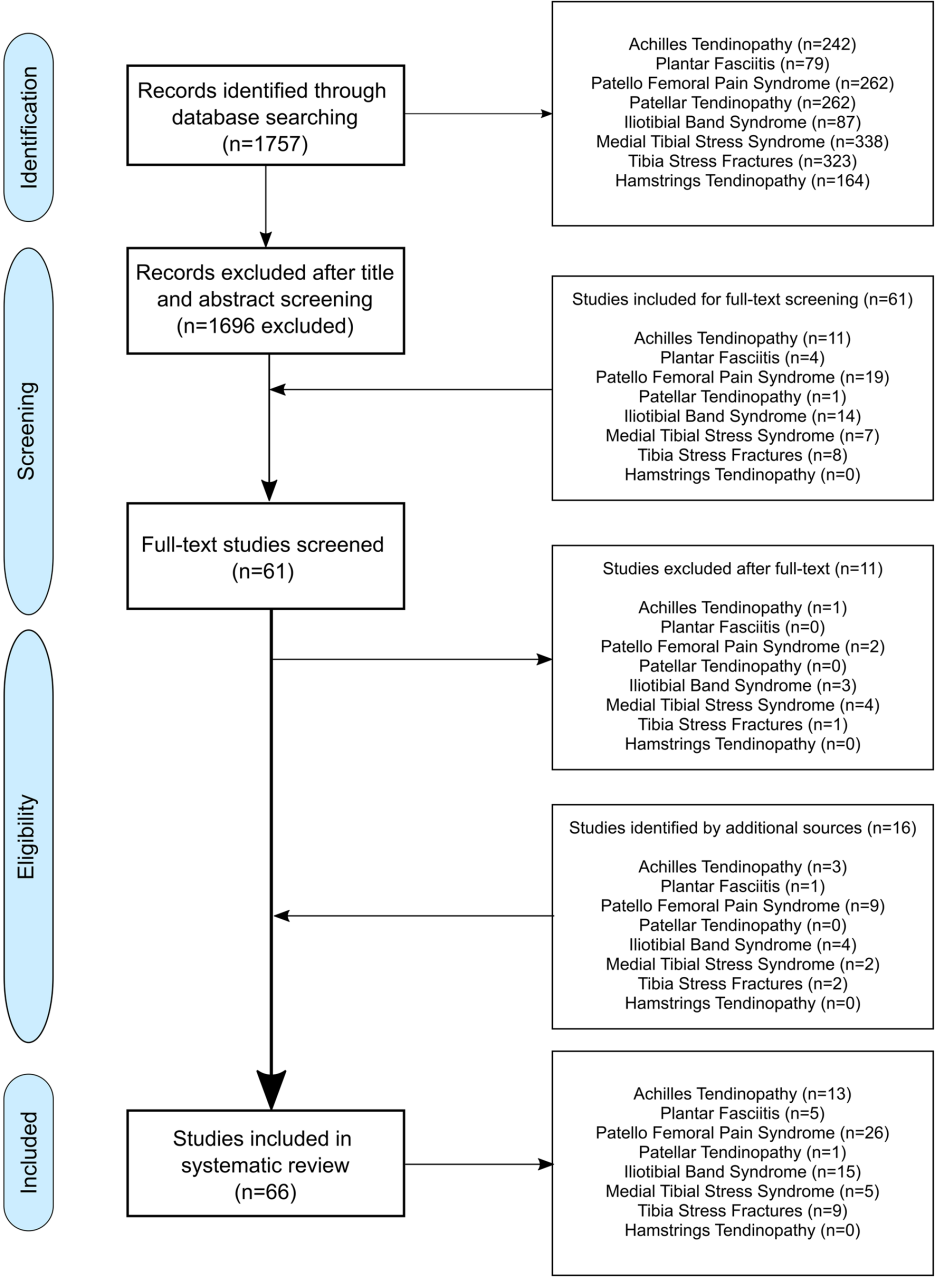
Flow-chart of the identification process. The numbers for articles per injury do not sum up to the total number of articles because some studies have addressed multiple running-related injuries

After identification, screening, and applying the exclusion and inclusion criteria, 66 articles were included in the review (Fig. 1).

**Fig. 2:**
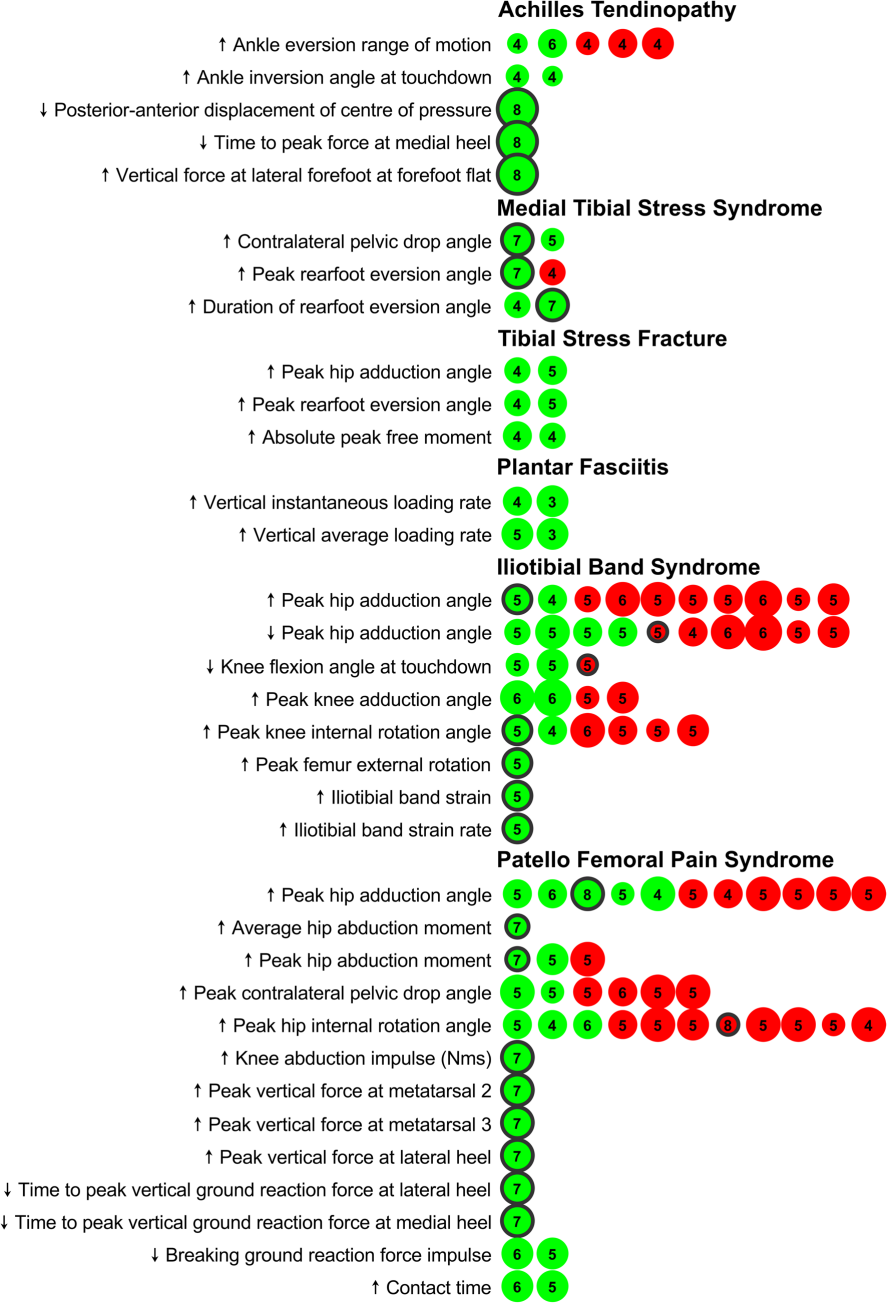
Graphic representation of the evidence associated with running-related risk factors that have passed our predefined relevance criterion (at least a significant difference in one prospective study or two retrospective studies). Dot size scales with Downs & Black quality rating of the studies (i.e., the bigger a dot, the higher the quality rating of the study). The number in the dots is the risk of bias score of the study. The green color represents a study that had found a significant difference between a group of injured runners compared to control. Red colors represent a study that could not find a significant difference between groups. Black circles around dots indicate a prospective study design (no circles = retrospective study design)

**Fig. 3:**
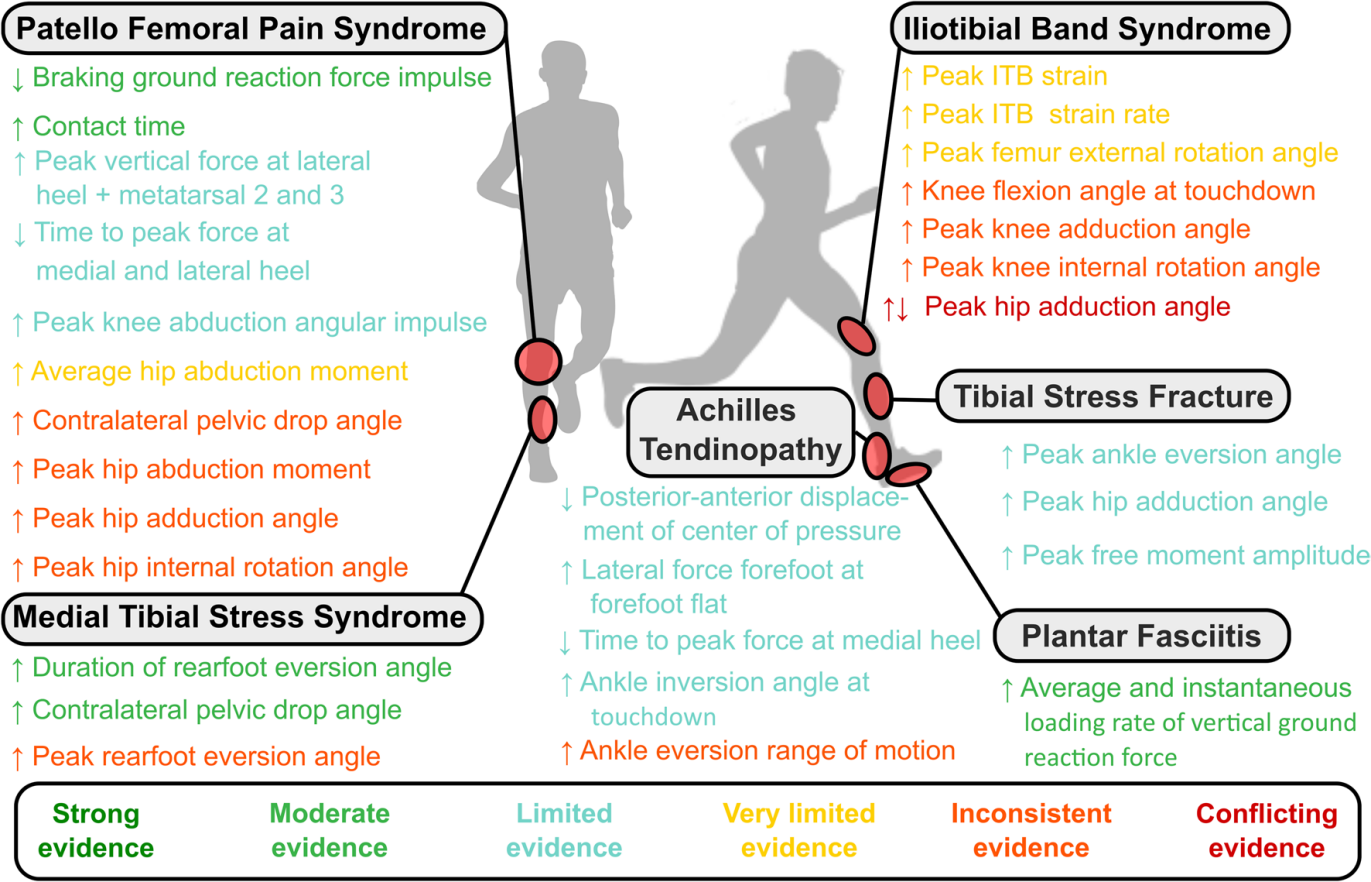
Overview of the evidence associated with running-related risk factors that have passed our predefined relevance criterion (at least a significant difference in one prospective study or two retrospective studies). *ITB* Iliotibial band. ↑ indicates that runners suffering from a running overuse injury had higher values of this biomechanical risk factor compared to non-injured runners. ↓ indicates that runners suffering from a running overuse injury had lower values of this biomechanical risk factor compared to non-injured runners. ↑↓ indicates conflicting evidence (i.e., at least one study showing higher and one study showing lower values of this biomechanical risk factor in injured compared to non-injured runners)

In the following, we report the findings independently for each ROI considered. The findings are summarized graphically in Figs. 2 and 3. Detailed results on study details (SDC3), quality assessment, and risk of bias rating (SDC2) can be found in the SDC.

### Achilles Tendinopathy (AT)

We identified 13 studies (twelve retrospective, one prospective) that had analyzed, in total, 123 different potential BRFs for AT (SDC4) through our systematic screening of the literature [20–32]. Out of these parameters, five BRFs were identified in either two independent retrospective studies or one prospective study, following our predefined relevance criterion.

Two BRFs related to the motion of the ankle joint in the frontal plane (rearfoot inversion-eversion relative to the tibia) were identified. Two medium quality retrospective studies (D&B: 7–10), one with a high risk of bias [risk of bias score (ROBS): 4], identified increased ankle range of motion from touchdown (i.e., initial contact of the foot with the ground) to maximum rearfoot eversion during the stance phase as a BRF [22, 30]. However, three retrospective studies (D&B: 8–11; ROBS: 4) [20, 25, 28] could not establish a difference in ankle eversion range of motion in runners with compared to runners without a history of AT (Fig. 2). Furthermore, more pronounced ankle inversion at initial contact with the ground was retrospectively identified as a BRF for AT by two medium quality studies with a high risk of bias (D&B: 7–8; ROBS: 4) [28, 30].

A high quality (D&B: 14) [21] prospective study with low risk of bias (ROBS: 8) using a pressure plate found that novice runners who developed AT within a 10-week follow-up period showed three differences in their plantar pressure application during the stance phase compared to novice runners who remained injury-free: first, a reduced antero-posterior displacement of the center of pressure during the stance phase; second, higher vertical forces applied through the lateral part of the forefoot at the instant of forefoot flat; third, a reduced time to peak force at the medial heel (Fig. 2).

Many additional parameters differed between runners suffering from AT and runners who did not. However, these results were only found in single retrospective studies and did not follow our predefined quality criterion. For a complete list of all parameters for all ROIs, please refer to SDC4.

In summary, we identified limited evidence for a reduced anterior–posterior displacement of the center of pressure, higher vertical forces applied through the lateral part of the forefoot at the instant of forefoot flat, and a reduced time to peak force at the medial heel during the stance phase as BRFs for AT. We further found limited evidence for increased ankle inversion angle at initial contact and inconsistent evidence for ankle eversion range of motion from initial contact to peak rearfoot eversion during stance as BRFs for AT (Fig. 3).

### Medial Tibial Stress Syndrome (MTSS)

Our search resulted in five (four retrospective, one prospective) studies addressing BRFs for MTSS [20, 32–35]. In these studies, 34 individual BRFs were investigated (SDC4). However, only three BRFs matched our relevance criterion.

In a high quality (D&B: 11; ROBS: 7) prospective study, competitive runners (NCAA Division 1) developing MTSS during a 2-year follow-up period ran with greater peak rearfoot eversion relative to the tibia, and their ankle joints remained in an everted position for a longer time during the stance phase compared to runners who did not develop MTSS [33]. Furthermore, in the same study, runners developing MTSS had a greater peak contralateral pelvic drop during the stance phase compared to runners not suffering from MTSS [33]. Higher peak contralateral pelvic drop was further identified by a moderate quality retrospective study with a high risk of bias (D&B: 8; ROBS: 5) [32]. The finding that runners with MTSS spend more time in eversion during stance was replicated in a moderate quality retrospective study (D&B: 9; ROBS: 4) [20]. However, the finding that peak eversion is a risk factor for MTSS was not replicated in this study [20] (Fig. 2).

In summary, we found moderate evidence for eversion time during stance, inconsistent evidence for peak eversion, and moderate evidence for peak contralateral pelvis drop during stance as BRFs for MTSS (Fig. 3).

### Tibial Stress Fractures (TSFs)

We identified nine retrospective studies addressing BRFs for TSFs [31, 36–43]. These studies considered 41 individual BRFs (SDC4). Three BRFs met our predefined relevance criterion. Two moderate quality studies (D&B: 9–10; ROBS: 4–5) found higher peak ankle eversion during stance for runners with a history of TSF [41, 43]. These same studies also reported greater peak hip adduction angles during stance for runners with a history of TSF compared to runners without a history of TSF [41, 43]. Further, two moderate quality studies (D&B: 9–10; ROBS: 4) found higher peak amplitudes of the free moment applied to the ground in runners with a history of TSF [40, 43].

One moderate quality retrospective study with a high risk of bias (D&B: 10; ROBS: 4) [39] found a statistically significant difference in peak tibial acceleration between runners with and without a history of TSF. Another moderate quality retrospective study with a high risk of bias (D&B: 9; ROBS: 4) [43] reported higher (Cohen's *d* = 0.3) peak tibial shock in runners with a history of injury. However, no direct statistical test for differences between the injured and non-injured groups was performed in this study. Therefore, by applying our definition of a BRF, we could not consider this result as evidence for peak tibial acceleration as a risk factor for TSF. Consequently, peak tibial shock did not pass our relevance criterion and is not reported in the summary figures (Figs. 2 and 3), and we did not quantify a level of evidence for this parameter.

We identified a similar situation when assessing the potential of the vertical ground reaction force's average and peak instantaneous loading rates as potential BRFs for TSF. Milner et al. 2006 (D&B: 10, ROBS: 4) [39] could identify both of these parameters as BRFs for TSF for female runners following our definition of a BRF. However, two other studies [43, 44] reported higher values for vertical loading rates in injured runners but did not test for statistical differences between groups. While there was another study (D&B: 11; ROBS: 3) [31] that failed to identify statistically significant differences in instantaneous and average vertical loading rate parameters between runners with a history of TSF compared to runners with no history of TSF, there are data highlighting a potential for vertical loading rate variables as BRFs for TSF, which is also reflected by a recent meta-analysis [45]. However, overall there was only one retrospective study showing a statistical difference between runners with and without a history of TSF in average and maximum instantaneous vertical loading rates [39]. Therefore, due to our strict relevance criterion and definition of BRFs, we did not report vertical loading rate parameters in the summary figures and did not determine a level of evidence for these parameters. However, we encourage researchers to address loading rate parameters in future studies.

In summary, we identified limited evidence for peak ankle eversion, peak hip adduction, and peak free moment amplitude as BRFs for TSF (Fig. 3).

### Plantar Fasciitis (PF)

Our search resulted in five retrospective studies considering 46 potential BRFs for PF [31, 46–49]. Two out of these 46 potential BRFs matched our predefined relevance criterion (SDC4). Runners with a PF history created higher instantaneous vertical loading rates of the ground reaction force in two retrospective studies [31, 48]. One study was of high quality (D&B: 11) but also with a high risk of bias (ROBS: 3) [31], while the other study was of moderate quality (D&B: 10) and also a high risk of bias (ROBS: 4) [48]. Further, two high-quality (D&B: 11; ROBS: 3–5) studies found that runners with PF history applied vertical forces at a higher average loading rate to the ground [31, 46].

In summary, we found moderate evidence for average and instantaneous vertical loading rates of the ground reaction force as BRFs for PF (Fig. 3).

### Iliotibial Band Syndrome (ITBS)

We found 15 studies (three prospective and 12 retrospective) considering 93 potential BRFs for ITBS (SDC4) [31, 32, 49–61]. Of these 93 potential BRFs, eight followed our relevance criterion. At the hip joint, conflicting evidence was found for the peak hip adduction angle. While one moderate quality retrospective study (D&B: 10; ROBS: 4) [56] and one moderate quality prospective study (D&B: 10; ROBS: 5) [59] found significantly higher peak hip adduction angles in runners with ITBS, three moderate (D&B: 9–10; ROBS: 5) [51, 53, 57] and one high quality (D&B: 12; ROBS: 5) [54] retrospective studies found reduced peak hip adduction angles during the stance phase in runners with ITBS compared to non-injured runners (Fig. 2).

In addition to the six studies [51, 53, 54, 56, 57, 59] showing conflicting evidence regarding peak hip adduction as a BRF for ITBS (Fig. 2), another four retrospective studies (D&B: 8–13; ROBS: 5–6) [32, 52, 54, 61] could not establish a difference (neither higher nor lower values) in maximum hip adduction in runners with compared to runners without a history of ITBS (Fig. 2).

A moderate quality (D&B: 10; ROBS: 5) prospective study found higher peak femoral external rotation (relative to the laboratory coordinate system) during stance in runners who developed ITBS compared to their control group [59] (Fig. 2).

At the knee, one moderate (D&B: 8; ROBS: 5) [32] and one high-quality (D&B: 11; ROBS: 5) [61] retrospective study identified reduced knee flexion angles at touchdown in runners with compared to runners without a history of ITBS (Fig. 2). However, these findings regarding knee flexion angles at touchdown could not be replicated by one moderate quality prospective (D&B: 10, ROBS: 5) study [59] (Fig. 2).

Further, a moderate quality retrospective study (D&B: 10; ROBS: 4) [56] and one moderate quality prospective study (D&B: 10; ROBS: 5) [59] found significantly higher peak knee internal rotation angles during the stance phase in runners with ITBS compared to non-injured runners. However, peak knee internal rotation was not identified as a risk factor for ITBS in four other retrospective studies (D&B: 8–12; ROBS: 5–6) [32, 53, 55, 61] (Fig. 2).

Further, two high-quality retrospective studies (D&B: 12–13; ROBS: 6) reported significantly higher peak knee adduction angles in runners with compared to runners without a history of ITBS [52, 55]. However, two retrospective studies (D&B: 8–11; ROBS: 5) [32, 61] failed to replicate the evidence concerning higher peak knee adduction angles as a BRF for ITBS (Fig. 2).

When applying a computer model which calculates the kinematics of the ITB, Hamill et al. (D&B: 10; ROBS: 5) [58] identified increased ITB strain and strain rates in runners with compared to runners without a history of ITBS (Fig. 2) within a prospective study.

In summary, our systematic review established inconsistent evidence for less knee flexion at touchdown, increased peak knee adduction angle, and peak knee internal rotation angle as BRFs for ITB. We found very limited evidence for increased ITB strain, increased ITB strain rate, and increased peak femur external rotation as BRFs for ITB. Further, we found conflicting evidence for peak hip adduction as a BRF for ITBS (Fig. 3).

### Patello Femoral Pain Syndrome (PFPS)

Twenty-six studies (four prospective and 22 retrospective) considering BRFs for PFPS were included in the systematic review [31, 32, 62–65, 65–84]. These studies analyzed in total 120 potential BRFs (SDC4). Of these, 13 BRFs matched our predefined relevance criterion.

At the hip, higher peak adduction angles were identified as risk factors by one high quality prospective study with low risk of bias (D&B: 11; ROBS: 8) [66], one high quality retrospective study with high risk of bias (D&B: 12; ROBS: 4) [84], and three moderate quality retrospective studies (D&B: 8–10; ROBS: 5–6) [32, 73, 76]. However, six other retrospective studies (D&B: 10–12; ROBS: 4–5) [63, 64, 69, 70, 80, 83] could not replicate the evidence for increased peak hip adduction angles as BRFs for PFPS (Fig. 2). A moderate quality prospective study with low risk of bias (D&B: 8; ROBS: 7) found increased average internal hip abduction moments in runners who developed PFPS compared to runners who did not [67] (Fig. 2). Further, one prospective (D&B: 8; ROBS: 7) [67] and one retrospective study (D&B: 11; ROBS: 5) [70] identified increased peak internal hip abduction moments as BRFs for PFPS (Fig. 2), even though this finding was not replicated in another retrospective study (D&B: 12; ROBS: 5) [80] (Fig. 2).

One high-quality (D&B: 12; ROBS: 5) [83] and one moderate quality study (D&B: 8; ROBS: 5) [32] identified higher peak contralateral pelvic drop in runners with compared to runners without a history of PFPS. However, four other retrospective studies (D&B: 10–12; ROBS: 5–6) [63, 73, 76, 80] could not identify a significant difference in peak contralateral pelvic drop angle between runners with and without a history of PFPS (Fig. 2). Furthermore, three moderate quality retrospective studies (D&B: 10, ROBS: 4–6) suggested that an increased peak hip internal rotation angle was associated with PFPS [64, 73, 76]. However, eight (one prospective, seven retrospective) studies (D&B: 8–12; ROBS: 4–8) [32, 63, 66, 69, 70, 80, 83, 84] could not replicate these results for increased peak hip internal rotation angle as a risk factor for PFPS (Fig. 2).

One high-quality (D&B: 11) prospective study with low risk of bias (ROBS: 7) found greater internal knee abduction impulse in runners developing PFPS compared to non-injured controls [71] (Fig. 2).

Several plantar pressure-related variables were identified by one high-quality (D&B: 11) prospective study with a low risk of bias (ROBS: 7) [74]. These were an increased peak vertical force at the lateral heel at touchdown (i.e., initial contact), as well as at the second and third metatarsal heads. Further, a reduced time to peak force at the medial and lateral heel was found (Fig. 2).

Two retrospective studies (D&B: 10–11; ROBS: 5–6) related a reduced braking impulse of the horizontal ground reaction force and a longer contact time with an increased risk for PFPS [75, 78] (Fig. 2).

In summary, we found moderate evidence for a reduced braking impulse of the ground reaction force and longer ground contact times as BRFs for PFPS. There was limited evidence for the above-mentioned plantar pressure-related parameters and increased internal knee abduction angular impulse. We found very limited evidence for increased average internal hip abduction moments. Finally, we identified inconsistent evidence for increased peak contralateral pelvic drop, increased peak hip adduction and internal rotation angles, as well as increased peak hip internal abduction moments during stance (Fig. 3).

### Patellar Tendinopathy (PT) and Hamstring Tendinopathy (HT)

A moderate quality study with a high risk of bias (D&B: 7; ROBS: 3) analyzed 42 potential BRFs for PT [85]. However, since this was the only study identified, our predefined relevance criterion was not met. We could not identify a study focussing on BRFs for HT.

## Discussion

This systematic review aimed to extract the evidence for BRFs for specific ROIs from the existing literature. While there are several important previous reviews on the role of BRFs for the development of running-related injuries, our work adds several relevant pieces to the complex puzzle of ROI development. It is the first systematic review that focuses on BRFs for the most prevalent ROIs while using the same inclusion and exclusion criteria for all considered injuries. Previous reviews either did not report overuse injuries for specific types of injuries [18] or focused on a single overuse injury [15, 86–94]. Further, some reviews focused only on prospective studies [18]. While these studies are superior in their strength of evidence to retrospective studies, the majority of research on BRFs for ROIs has used retrospective designs. By applying our relevance criterion (potential BRFs identified from at least two retrospective studies or one prospective study), we acknowledged the superior evidence of the prospective study design while at the same time including insight gained from retrospective studies.

### Limitations

Despite the several strengths of this work, we need to highlight several limitations. Due to the relatively low numbers of studies for certain BRFs and the lack of results reported or analyzed in the considered studies, we could not differentiate our findings for different groups of runners. Different runners likely vary in their structure-specific stress tolerance levels and adaptation. In addition, running mechanics differ between runners, for example between males and females [95]. Sex differences have also been observed between male and female runners suffering, for example from PFPS [83, 84], further emphasizing the need for considering individual factors in greater detail in future studies addressing BRFs for ROIs. Therefore, we recommend that future studies on BRFs should report as many details of the running population as possible.

Pooling together the findings from all studies addressing an ROI without considering relevant covariates as we did in this review might identify inconclusive results. For example, increased peak hip adduction angles were only identified in female runners [56, 59], not in studies including male runners.

Further, different studies used heterogeneous definitions of injury, types of runners (e.g., competitive vs. recreational), and outcome measures in the included full-text articles, which challenged comparison across studies. Also, most studies did not consider running volume in their assessment of injury risk between groups (e.g., incidence per 1000 h of running [2]) or tried to quantify workload characteristics by other means. Since increased training volume or intensity likely amplify the risk associated with specific running patterns, a lack of control concerning training characteristics between injured and non-injured groups of runners might result in misleading findings for BRFs.

This review focused on the ROIs reported by Lopes et al. [12]. Future studies might extend our approach to more ROIs, e.g., femoral and metatarsal stress fractures, to provide a complete picture of BRFs for the wide variety of ROIs.

Further, we only used PubMed as our primary search database. Nonetheless, we felt that using PubMed with a relatively broad search strategy was most relevant for the review, and we performed additional searches using connected papers and included papers identified within the reference lists. Therefore, we believe that the chances of missing relevant papers should be minimal.

### Outlook

Running injuries occur within a complex interaction between the stresses applied to body tissues while running, individual factors (e.g., age, sex, previous injuries), training (e.g., intensity, volume, rest intervals), and lifestyle (e.g., nutrition, sleep) factors. Developing a holistic injury risk profile of a runner should consider these individual factors.

A list of relevant BRFs can inform the creation of a running biomechanics-related risk profile as one piece in this complex puzzle. Such a BRF profile considers the ROI risk due to the way somebody is running. Based on such a running-related risk profile, individualized footwear could be developed, or footwear might be reconsidered to change the running biomechanics towards a less risky profile. Considering specific injuries is significant progress for injury risk profiling. For example, running shoes can be designed to shift loading between musculoskeletal structures in the lower extremity and hence specifically address injury-specific risk factors [5]. A BRF profile might also inform prevention training programs to strengthen biological tissues at risk or help to develop feedback tools that facilitate running gait retraining towards a less pronounced risk profile [96].

Gait retraining can modify several biomechanical variables identified within this review [97, 98]. Based on this review, reducing loading rates could be a strategy to reduce the risk for PF and potentially TSF. In a study with novice runners who underwent gait-retraining to reduce loading rates [98], lower injury incidences overall and particularly for PT were found in the gait-retraining compared to a non-gait-retraining control group. This example highlights the potential of gait retraining based on a BRF profile to reduce injury risk.

However, the data presented in this review are not sufficient to create meaningful running related-risk profiles at the moment. This is for several reasons.

First, there is clearly not enough evidence in support of BRFs existing in the literature. Larger, high-quality, prospective studies need to be performed to resolve this issue in the future. In this review, we identified a large variety of methodological details (SDC 3), which makes the pooling of findings for a more holistic understanding difficult. Future approaches should aim to standardize experimental protocols and data analysis methods to overcome these difficulties. Further, only a few studies considered the fatigue response in running mechanics when addressing BRFs. Since ROIs result from repetitive loading over time, we suggest improved integration of changes due to running-induced fatigue in the assessment of BRFs.

Further, studies need to consider the interaction between BRFs and other factors modulating injury risk. Knowledge of potential interactions might enable the weighting of BRFs to emphasize specific changes in running mechanics to reduce injury risk. For example, having suffered from a previous injury is a well-accepted, non-running related injury risk factor [99]. Consequently, knowledge of the injury history could be used to increase the weighting for BRFs related to the specific ROIs experienced by a runner in the past. Consequently, these BRFs could be considered more in individualized running shoe design, prevention training protocols, or running gait feedback tools.

Finally, there is a general lack of validation of BRFs in the published literature. With only a few exceptions (e.g. [98, 98]), no intervention studies have been performed that have first changed a BRF through gait retraining or footwear interventions and subsequently quantified reductions in ROI severity.

Based on these considerations, the findings of this review, and recent injury development frameworks [6, 7, 11], we propose the following directions for future research. These directions can be broadly categorized by either using larger datasets with potentially lower data precision or smaller datasets with higher precision.

The big data macroscopic approach can leverage the recent developments in wearable sensor technology and artificial intelligence. Today, running movement data can be captured during every training session and uploaded to large databases. However, the insight gained from the big data approach relies on the ability to determine relevant features (i.e., functional or discrete features related to injury risk) from these sensor signals, potentially with the assistance of artificial intelligence. The parameters identified from this review can serve as a starting point for such a data exploration. However, this approach will only be successful if BRFs can be measured with high validity and repeatability within the running community.

Tools to collect and store data on large scales while using user feedback to label the occurrence of running-related pain or injuries might allow further insight by considering not only single data collection sessions but, in principle, the entire training history of an individual (e.g., changes associated with fatigue) [100–102]. Further running mechanics and training characteristics might be considered in their interaction with other individual or lifestyle-related factors. Research collaborations that use the same data capture and labeling methodology seem ideally suited to solve this task. Larger research collaborations might further present a solution to limitations that were present in most of the studies identified by this review, for example small sample sizes, a lack of considering confounding variables, application of different methodologies to determine running mechanics, and different definitions of injuries.

As identified by this review, the current body of literature on BRFs for ROIs is dominated by retrospective studies, rather than prospective studies (Fig. 2). From retrospective studies, causation between BRFs and injury cannot be inferred directly. High-quality, large-scale prospective studies are required to identify BRFs for ROIs. In conjunction with these studies, higher resolution analyses to quantify the loading experienced by specific tissues in distance running will also be of benefit to understand the pathomechanics underpinning running overuse injuries.

Such small data microscopic approaches rely on improvements in biomechanical modeling approaches that can improve our understanding of how running biomechanics are linked to the stress of the tissues involved in ROIs. Here, the combination of individualized musculoskeletal models with, for example, finite element models of the tissues under consideration seems to offer the potential for improved targeting of runner populations at risk and increased understanding of cause-effect relationships in ROI development. Single subjects study designs applying very detailed modeling techniques might further improve our understanding of injury development since the etiology of an injury is not the same for all patients diagnosed with the same injury. However, currently, these techniques are time-consuming and rely on many assumptions that challenge the validity of the calculated stress characteristics. Therefore, the discipline of biomechanics should also target a more efficient yet precise quantification of input variables for these model calculations.

Finally, validation studies that apply interventions to reduce BRFs for specific injuries should be performed to improve the understanding of cause-effect relationships and improve our understanding of the effectiveness of interventions derived from individual injury risk profiles.

BRFs should not be addressed in isolation, but rather in the context of relevant covariates in a comprehensive injury development framework [11]. A consensus on the minimum number and type of such framework variables for running injury research seems urgently needed to face this challenge.

## Conclusion

In summary, this is the first systematic review that summarises the evidence for BRFs for specific ROIs using the same search strategy and exclusion and inclusion criteria. Hopefully, this work can serve as the basis to identify runners at risk for specific ROIs and, from this basis, improve decisions on footwear design or use, training and rehabilitation programs, and sensor-based devices to monitor and improve individual running biomechanics.

## Supplementary Information

Below is the link to the electronic supplementary material.

Supplementary file1 (PDF 110 kb)

Supplementary file2 (XLSX 24 kb)

Supplementary file3 (XLSX 75 kb)

Supplementary file4 (XLSX 123 kb)

## Declarations

### Availability of data and material

All extracted information can be found in the figures and supplementary materials.
