# Supplementary material for: Running-Related Biomechanical Risk Factors for Overuse Injuries in Distance Runners: A Systematic Review Considering Injury Specificity and the Potentials for Future Research
Source: Sports Med. 2022 Mar 5;52(8):1863–77. doi: 10.1007/s40279-022-01666-3 (PMC9325808; doi:10.1007/s40279-022-01666-3)
Supplement: Supplementary file 1 — Supplementary file1 (PDF 110 kb) [file 40279_2022_1666_MOESM1_ESM.pdf]

Supplementary Materials – Search Strings

Running related biomechanical risk factors for overuse injuries in distance runners: A systematic review considering injury specificity and the potentials for future research

Steffen Willwacher[1,3*], Markus Kurz[2], Johanna Robbin[1,3], Matthias Thelen[3], Joseph Hamill[4], Luke Kelly[5], Patrick Mai[3]

[1]Department for Mechanical and Process Engineering, Offenburg University of Applied Sciences, Offenburg, Germany

[2]Department of Quality Technology & Mechanical Engineering, Mid Sweden University, Östersund, Sweden

[3]Institute for Biomechanics and Orthopaedics, German Sport University Cologne, Cologne, Germany

[4]Biomechanics Laboratory, University of Massachusetts, Amherst, MA, USA

[5]School of Human Movement and Nutrition Sciences, University of Queensland, St. Lucia, Queensland, Australia

Search Strings:

Achilles tendinopathy

Date of Access: 5.2.2021: N = 242

(runn*) AND (injur* OR risk OR factor* OR tend* OR syndrome OR stress* OR fasc* OR pain) AND (prospective OR retrospective OR cross* OR long* OR follow* OR case* OR cohort*) AND (achil*) NOT ("addresses"[Publication Type] OR "bibliography"[Publication Type] OR "biography"[Publication Type] OR "case reports"[Publication Type] OR "clinical conference"[Publication Type] OR "comment"[Publication Type] OR "congresses"[Publication Type] OR "dictionary"[Publication Type] OR "directory"[Publication Type] OR "editorial"[Publication Type] OR "festschrift"[Publication Type] OR "government publications"[Publication Type] OR "interview"[Publication Type] OR "lectures"[Publication Type] OR "legal cases"[Publication Type] OR "legislation"[Publication Type] OR "letter"[Publication Type] OR "news"[Publication Type] OR "newspaper article"[Publication Type] OR "retracted publication"[Publication Type] OR "retraction of publication"[Publication Type] OR "review"[Publication Type] OR "review literature"[Publication Type] OR "review of reported cases"[Publication Type] OR "review, academic"[Publication Type] OR "review, multicase"[Publication Type] OR "review, tutorial"[Publication Type] OR "scientific integrity review"[Publication Type] OR "technical report"[Publication Type] OR "twin study"[Publication Type] OR "validation studies"[Publication Type]) AND "Humans"[Species]

iliotibial band friction

Date of Access: 4.2.2021: N = 87

(runn*) AND (injur* OR risk OR factor* OR tend* OR syndrome OR stress* OR fasc* OR pain OR friction) AND (prospective OR retrospective OR cross* OR long* OR follow* OR case*) AND (ilio*) NOT ("addresses"[Publication Type] OR "bibliography"[Publication Type] OR "biography"[Publication Type] OR "case reports"[Publication Type] OR "clinical conference"[Publication Type] OR "comment"[Publication Type] OR "congresses"[Publication Type] OR

“dictionary”[Publication Type] OR “directory”[Publication Type] OR “editorial”[Publication Type] OR “festschrift”[Publication Type] OR “government publications”[Publication Type] OR “interview”[Publication Type] OR “lectures”[Publication Type] OR “legal cases”[Publication Type] OR “legislation”[Publication Type] OR “letter”[Publication Type] OR “news”[Publication Type] OR “newspaper article”[Publication Type] OR “retracted publication”[Publication Type] OR “retraction of publication”[Publication Type] OR “review”[Publication Type] OR “review literature”[Publication Type] OR “review of reported cases”[Publication Type] OR “review, academic”[Publication Type] OR “review, multicase”[Publication Type] OR “review, tutorial”[Publication Type] OR “scientific integrity review”[Publication Type] OR “technical report”[Publication Type] OR “twin study”[Publication Type] OR “validation studies”[Publication Type]) AND “Humans”[Species]

Medial tibial stress syndrome

Date of Access: 4.2.2021: N = 338

(runn*) AND (injur* OR risk OR factor* OR tend* OR syndrome OR stress* OR fasc* OR pain OR femu* OR frac* OR medial) AND (prospective OR retrospective OR cross* OR long* OR follow* OR cohort) AND (tibia*) NOT (“addresses”[Publication Type] OR “bibliography”[Publication Type] OR “biography”[Publication Type] OR “case reports”[Publication Type] OR “clinical conference”[Publication Type] OR “comment”[Publication Type] OR “congresses”[Publication Type] OR “dictionary”[Publication Type] OR “directory”[Publication Type] OR “editorial”[Publication Type] OR “festschrift”[Publication Type] OR “government publications”[Publication Type] OR “interview”[Publication Type] OR “lectures”[Publication Type] OR “legal cases”[Publication Type] OR “legislation”[Publication Type] OR “letter”[Publication Type] OR “news”[Publication Type] OR “newspaper article”[Publication Type] OR “retracted publication”[Publication Type] OR “retraction of publication”[Publication Type] OR “review”[Publication Type] OR “review literature”[Publication Type] OR “review of reported cases”[Publication Type] OR “review, academic”[Publication Type] OR “review, multicase”[Publication Type] OR “review, tutorial”[Publication Type] OR “scientific integrity review”[Publication Type] OR “technical report”[Publication Type] OR “twin study”[Publication Type] OR “validation studies”[Publication Type]) AND “Humans”[Species]

Tibia stress fracture

Date of Access: 5.2.2021: N = 323

(runn*) AND (injur* OR risk OR factor* OR tend* OR syndrome OR stress* OR fasc* OR pain OR femu* OR frac*) AND (prospective OR retrospective OR cross* OR long* OR follow* OR cohort) AND (tibia*) NOT (“addresses”[Publication Type] OR “bibliography”[Publication Type] OR “biography”[Publication Type] OR “case reports”[Publication Type] OR “clinical conference”[Publication Type] OR “comment”[Publication Type] OR “congresses”[Publication Type] OR “dictionary”[Publication Type] OR “directory”[Publication Type] OR “editorial”[Publication Type] OR “festschrift”[Publication Type] OR “government publications”[Publication Type] OR “interview”[Publication Type] OR “lectures”[Publication Type] OR “legal cases”[Publication Type] OR “legislation”[Publication Type] OR “letter”[Publication Type] OR “news”[Publication Type] OR “newspaper article”[Publication Type] OR “retracted publication”[Publication Type] OR “retraction of publication”[Publication Type] OR “review”[Publication Type] OR “review literature”[Publication Type] OR “review of reported cases”[Publication Type] OR “review, academic”[Publication Type] OR “review, multicase”[Publication Type] OR “review, tutorial”[Publication Type] OR “scientific integrity review”[Publication Type] OR “technical report”[Publication Type] OR “twin study”[Publication Type] OR “validation studies”[Publication Type]) AND “Humans”[Species]

Plantar fasciitis

Date of Access: 5.2.2021: N = 79

(runn*) AND (injur* OR risk OR factor* OR tend* OR syndrome OR stress* OR fasc* OR pain) AND (prospective OR retrospective OR cross* OR long* OR follow* OR case* OR cohort*) AND ((plant* AND fasc*) OR (heel* AND spur*)) NOT (“addresses”[Publication Type] OR “bibliography”[Publication Type] OR “biography”[Publication Type] OR “case reports”[Publication Type] OR “clinical conference”[Publication Type] OR “comment”[Publication Type] OR “congresses”[Publication Type] OR “dictionary”[Publication Type] OR “directory”[Publication Type] OR “editorial”[Publication Type] OR “festschrift”[Publication Type] OR “government publications”[Publication Type] OR “interview”[Publication Type] OR “lectures”[Publication Type] OR “legal cases”[Publication Type] OR “legislation”[Publication Type] OR “letter”[Publication Type] OR “news”[Publication Type] OR “newspaper article”[Publication Type] OR “retracted publication”[Publication Type] OR “retraction of publication”[Publication Type] OR “review”[Publication Type] OR “review literature”[Publication Type] OR “review of reported cases”[Publication Type] OR “review, academic”[Publication Type] OR “review, multicase”[Publication Type] OR “review, tutorial”[Publication Type] OR “scientific integrity review”[Publication Type] OR “technical report”[Publication Type] OR “twin study”[Publication Type] OR “validation studies”[Publication Type]) AND “Humans”[Species]

Patellofemoral pain syndrome

Date of Access: am 5.2.2021: N = 262

(runn*) AND (injur* OR risk OR factor* OR tend* OR syndrome OR stress* OR fasc* OR pain OR femo* OR femu*) AND (prospective OR retrospective OR cross* OR long* OR follow* OR cohort) AND (patel*) NOT (“addresses”[Publication Type] OR “bibliography”[Publication Type] OR “biography”[Publication Type] OR “case reports”[Publication Type] OR “clinical conference”[Publication Type] OR “comment”[Publication Type] OR “congresses”[Publication Type] OR “dictionary”[Publication Type] OR “directory”[Publication Type] OR

“editorial”[Publication Type] OR “festschrift”[Publication Type] OR “government publications”[Publication Type] OR “interview”[Publication Type] OR “lectures”[Publication Type] OR “legal cases”[Publication Type] OR “legislation”[Publication Type] OR “letter”[Publication Type] OR “news”[Publication Type] OR “newspaper article”[Publication Type] OR “retracted publication”[Publication Type] OR “retraction of publication”[Publication Type] OR “review”[Publication Type] OR “review literature”[Publication Type] OR “review of reported cases”[Publication Type] OR “review, academic”[Publication Type] OR “review, multicase”[Publication Type] OR “review, tutorial”[Publication Type] OR “scientific integrity review”[Publication Type] OR “technical report”[Publication Type] OR “twin study”[Publication Type] OR “validation studies”[Publication Type]) AND “Humans”[Species]

Patellar tendinopathy Jumpers knee

Date of Access: 5.2.2021: N = 262

(runn*) AND (injur* OR risk OR factor* OR tend* OR syndrome OR stress* OR fasc* OR pain OR femo* OR femu*) AND (prospective OR retrospective OR cross* OR long* OR follow* OR cohort) AND (patel* OR (jump* AND knee*)) NOT (“addresses”[Publication Type] OR “bibliography”[Publication Type] OR “biography”[Publication Type] OR “case reports”[Publication Type] OR “clinical conference”[Publication Type] OR “comment”[Publication Type] OR “congresses”[Publication Type] OR “dictionary”[Publication Type] OR “directory”[Publication Type] OR “editorial”[Publication Type] OR “festschrift”[Publication Type] OR “government publications”[Publication Type] OR “interview”[Publication Type] OR “lectures”[Publication Type] OR “legal cases”[Publication Type] OR “legislation”[Publication Type] OR “letter”[Publication Type] OR “news”[Publication Type] OR “newspaper article”[Publication Type] OR “retracted publication”[Publication Type] OR “retraction of publication”[Publication Type] OR “review”[Publication Type] OR “review literature”[Publication Type] OR “review of reported cases”[Publication Type] OR “review, academic”[Publication Type] OR “review, multicase”[Publication Type] OR “review, tutorial”[Publication Type] OR “scientific integrity review”[Publication Type] OR “technical report”[Publication Type]

OR “twin study”[Publication Type] OR “validation studies”[Publication Type]) AND “Humans”[Species]

Hamstring tendinopathy

Date of Access: 5.2.2021: N = 164

(runn*) AND (injur* OR risk OR factor* OR tend* OR syndrome OR stress* OR fasc* OR pain) AND (prospective OR retrospective OR cross* OR long* OR follow* OR case* OR cohort) AND (hamstring*) NOT ("addresses"[Publication Type] OR "bibliography"[Publication Type] OR "biography"[Publication Type] OR "case reports"[Publication Type] OR "clinical conference"[Publication Type] OR "comment"[Publication Type] OR "congresses"[Publication Type] OR "dictionary"[Publication Type] OR "directory"[Publication Type] OR "editorial"[Publication Type] OR "festschrift"[Publication Type] OR "government publications"[Publication Type] OR "interview"[Publication Type] OR "lectures"[Publication Type] OR "legal cases"[Publication Type] OR "legislation"[Publication Type] OR "letter"[Publication Type] OR "news"[Publication Type] OR "newspaper article"[Publication Type] OR "retracted publication"[Publication Type] OR "retraction of publication"[Publication Type] OR "review"[Publication Type] OR "review literature"[Publication Type] OR "review of reported cases"[Publication Type] OR "review, academic"[Publication Type] OR "review, multicase"[Publication Type] OR "review, tutorial"[Publication Type] OR "scientific integrity review"[Publication Type] OR "technical report"[Publication Type] OR "twin study"[Publication Type] OR "validation studies"[Publication Type]) AND "Humans"[Species]
